# Supramolecular Interactions Induce Unexpectedly Strong Emissions from Triphenylamine-Functionalized Polytyrosine Blended with Poly(4-vinylpyridine)

**DOI:** 10.3390/polym9100503

**Published:** 2017-10-12

**Authors:** Yu-Ru Jheng, Mohamed Gamal Mohamed, Shiao-Wei Kuo

**Affiliations:** 1 Department of Materials and Optoelectronic Science, National Sun Yat-Sen University, Kaohsiung 80424, Taiwan; m043100015@student.nsysu.edu.tw (Y.-R.J.); mgamal.eldin12@yahoo.com (M.G.M.); 2 Ningbo Institute of Material Technology and Engineering, Key Laboratory of Graphene Technologies and Applications of Zhejiang Province, Chinese Academy of Science, Zhongguan West Road 1219, Ningbo 315201, China; 3 Department of Medicinal and Applied Chemistry, Kaohsiung Medical University, Kaohsiung 80424, Taiwan

**Keywords:** Supramolecular Interaction, polypeptides, aggregation-induced emission, polymer blend, secondary structure

## Abstract

In this study, we synthesized a triphenylamine-functionalized polytyrosine (PTyr-TPA) through living ring opening polymerization with 4,4′-diamino-4″-methoxytriphenylamine (TPA-NH_2_) as an initiator, and used Fourier transform infrared (FTIR) and nuclear magnetic resonance spectroscopy to confirm the chemical structure. Photoluminescence spectroscopy revealed the photophysical properties of TPA-NH_2_ and PTyr-TPA and suggested that TPA-NH_2_ exhibited aggregation-caused quenching; in contrast, attaching the initiator to the rigid rod conformation of the PTyr segments caused PTyr-TPA to display aggregation-induced emission behavior. Differential scanning calorimetry revealed single glass transition temperatures for miscible PTyr-TPA/P4VP blends, the result of intermolecular hydrogen bonding between the pyridine units of P4VP and the phenolic OH units of PTyr-TPA, as confirmed through FTIR spectroscopic analyses. Furthermore, the chain behavior of PTyr-TPA transformed from a β-sheet conformation to random coils after blending with P4VP, as determined using wide-angle X-ray diffraction. These findings suggest that the decreased emission intensity of PTyr-TPA resulted from release of the restricted intramolecular rotation of the triphenylamine moiety in the polypeptide center.

## 1. Introduction

Amino acid polymers (e.g., polypeptides) perform many important roles because they can form stable well-defined secondary structures, including α-helical and β-sheet conformations, both in the solid state and in solution [1,2,3,4,5]. The β-sheet conformation is stabilized through intermolecular hydrogen bonding, whereas the α-helical conformation is stabilized by intramolecular hydrogen bonding that can lead to the formation of rigid-rod-like polymers [6,7,8]. Because of their potential applications, polypeptides have been studied widely; they can be prepared readily through ring-opening living polymerization of *N*-carboxyanhydride (NCA) monomers. In general, the conjugation of polypeptides to functional moieties or polymer segments, e.g., poly(ethylene oxide) [9,10,11], poly(ε-caprolactone) [12], poly(2-ethyl-2-oxazoline) [13], pyrene [14,15], and polyhedral oligomeric silsesquioxane (POSS) nanoparticles [16,17,18,19,20], can result in materials exhibiting attractive properties (e.g., thermoresponsive behavior and molecular recognition capabilities).

In addition to conjugating functional moieties or polymer segments into the side chains or main chains of polypeptides, blending with other polymers is another approach toward mediating the self-assembly behavior or secondary structures of polypeptides [21,22,23]. For instance, we investigated the secondary structural conformations of poly(γ-benzyl-l-glutamate) (PBLG) after its blending with random coil homopolymers; the miscibility and conformational behavior were strongly dependent on the strength of the hydrogen bonding interactions [24,25]. In addition, we also found that the secondary structural conformations of polytyrosine (PTyr) in different solutions were affected by blending with poly(4-vinylpyridine) (P4VP) [26,27,28]. The α-helical and β-sheet conformations of PTyr allowed it to form aggregated chains in CH_3_OH solution and separated random coils in DMF solution, as determined using wide-angle X-ray diffraction (WAXD) and Fourier transform infrared (FTIR) spectroscopy [26].

Fluorescent organic compounds have received much attention because of their potential applications in [29,30,31], for example, organic lasers [32], fluorescent biological labels [33], and fluorescence sensors [31]. Most traditional organic fluorophores (e.g., carbazolyl, pyrene, and dansyl moieties) possess large delocalized π-conjugated units; they emit strongly in dilute solution, but more weakly at higher concentrations or in the solid state—a phenomenon known as aggregation-caused quenching (ACQ) arising from aggregation of their planar conjugated aromatic rings [34]. This phenomenon can result in increased π-stacking and, therefore, the formation of excimers and exciplexes [35,36,37,38]. As a result, a challenge remains to develop new fluorescent compounds that emit as efficiently in the aggregated state as they do in solution. Tang et al. prepared the first aggregation-induced emission (AIE) materials based on the pentaphenyl derivatives of silole; these materials exhibited weaker fluorescence in solution and higher fluorescence in the aggregated state [39,40,41]. They also proposed that this AIE mechanism was correlated with a restriction of intramolecular rotation (RIR) mechanism for chromophoric compounds [39,40,41]. Furthermore, we have attached AIE compounds to polypeptides through covalent bonding, ionic interactions, and simple blending [42]. For instance, we synthesized tetraphenylthiophene (TP) displaying AIE properties and positioned it at the center or terminus of PBLG-based polypeptides [42]. We observed that the TP unit at the center of PBLG functioned as steric blocks, due to the α-helical conformation of PBLG peptide chain, and minimized the AIE behavior. Recently, Hong et al. prepared blends of 2-(anthracen-9-yl)vinylpyridine (AnPy) and PTyr, stabilized through intermolecular hydrogen bonding, that displayed intramolecular charge transfer and AIE behavior [43]. In this present study, we employed 4,4′-diamino-4″-methoxytriphenylamine (TPA-NH_2_) as an initiator for the preparation of PTyr-TPA through ring-opening living polymerization of an l-tyrosine-*N*-carboxyanhydride monomer. We used photoluminescence (PL) spectroscopy to examine the fluorescence properties of TPA-NH_2_ and PTyr-TPA. We then employed differential scanning calorimetry (DSC), FTIR spectroscopy, WAXD, and PL spectroscopy to examine the miscibility, hydrogen bonding, fluorescence, and conformational behavior of PTyr-TPA/P4VP blends, prepared through simple blending of PTyr-TPA with P4VP in DMF.

## 2. Experimental

### 2.1. Materials

P4VP (Aldrich, St. Louis, MO, USA, *M*_n_ = 160,000 g/mol), triphosgene (TCI, Tokyo, Japan), l-tyrosine (MP Biomedicals, Santa Ana, CA, USA), hexane (Arcos, Taipei, Taiwan, 99%), MeCN (Acros, Taipei, Taiwan, 99.5%), acetone (Acros, Taipei, Taiwan, 99.6%), dichloromethane (Acros, Taipei, Taiwan, 99%), MeOH (Acros, Taipei, Taiwan, 99.9%), dimethylsulfoxide (DMSO, Taipei, Taiwan, 99.7%), ethanol (Merck, Taipei, Taiwan, 99%), DMF (Merck, Taipei, Taiwan, 99%), and tetrahydrofuran (THF, Tedia, Taipei, Taiwan, 99%) were purchased commercially, while 10 wt % Pd/C, 1-fluoro-4-nitrobenzene (99%), and *p*-anisidine (99%) were purchased from Acros (Taipei, Taiwan) and used as received.

#### 2.1.1. 4,4′-Dinitro-4″-Methoxytriphenylamine

This synthesis was modified from a procedure reported in literature [44], using a 250 mL flask with a stirrer bar under a N_2_ atmosphere. *p*-Anisidine (2.93 g, 0.240 mol) and K_2_CO_3_ (17.9 g, 0.130 mol) were stirred in dry DMSO (50 mL) under a N_2_ atmosphere for 30 min and then 1-fluoro-4-nitrobenzene (6.80 g, 0.480 mol) was added. The mixture was then stirred at 150 °C for 48 h, resulting in the formation of an orange precipitate. The reaction mixture was cooled to room temperature and the crude product precipitated in cold water (200 mL). The precipitated powder was filtered off and washed three times with cold water. Drying under vacuum at 50 °C gave an orange powder. FTIR (KBr, cm^−1^): 1336, 1599 (NO_2_ stretch). ^1^H NMR (500 MHz, DMSO-*d*_6_, δ, ppm, coupling constant, *J*, Hz): 8.17 (4H, *J* = 9 Hz, NO_2_CCH), 7.21 (2H, *J* = 9 Hz OCH_3_CCH), 7.17 (4H, *J* = 9 Hz, NCCH), 7.04 (2H, *J* = 9 Hz, NCCH), 2.52 (3H, OCH_3_). ^13^C NMR (125 MHz, DMSO-*d*_6_, δ, ppm): 158.83, 152.19, 142.79, 142.421, 137.636, 137.224, 129.72, 126.16, 126.06, 122.31, 116.416, 55.693.

#### 2.1.2. 4,4′-Diamino-4″-Methoxytriphenylamine

In a modified version of a procedure reported in the literature [44], 4,4′-dinitro-4″-methoxytriphenylamine (7.34 g, 0.0200 mol) and Pd/C (0.13 g) were mixed under a N_2_ atmosphere in EtOH (100 mL) in a 200 mL flask equipped with the stirrer bar. The suspension was heated under reflux at 80 °C for 1 h, and then N_2_H_4_ (7 mL) was added dropwise slowly. The reaction mixture was heated for 24 h and then filtered to remove the Pd/C catalyst. After cooling, the precipitate was filtered off and vacuum-dried to obtain a gray product. FTIR (cm^−1^): 3449 and 3331 (N–H stretch). ^1^H NMR (500 MHz, DMSO-*d*_6_, δ, ppm, coupling constant, *J*, Hz): 6.47 (2H, *J* = 9 Hz, NH_2_CCH), 6.68 (2H, *J* = 9 Hz, NCCH), 6.69–6.73 (2H, *J* = 9 Hz, OCH_3_CCHNCCH), 4.82 (4H, NH_2_), 3.65 (3H, OCH_3_). ^13^C NMR (125 MHz, DMSO-*d*_6_, δ, ppm): 153.93, 145.30, 143.52, 137.99, 126.152, 121.53, 115.154, 55.212.

#### 2.1.3. Tyr-NCA Monomer

Using a previously reported procedure [26], a solution of triphosgene (2.65 g, 8.85 mmol) in MeCN (20 mL) was added dropwise to a solution of l-tyrosine (4.00 g, 22.1 mmol) in MeCN (350 mL) and then the mixture was heated under reflux (70 °C) under a N_2_ atmosphere in a 250 mL flask. After 5 h, the solution was cooled to 0 °C in an ice bath. The solvent was removed in a rotary evaporator and the residue recrystallized six times from THF/hexane to yield a pure white powder (2.8 g, 73%). FTIR (cm^−1^): 3306 (NH), 1841 (C=O stretching), 1776 (C=O), 1592, 751, 713 (Ar). ^1^H NMR (500 MHz, DMSO-*d*_6_, δ, ppm, coupling constant, *J*, Hz): 2.90 (d, *J* = 3.8 Hz, 2H, CH_2_), 4.69 (t, *J* = 5 Hz, 1H), 6.69 (d, *J* = 9 Hz, 2H, Ar), 9.02 (s, 1H, NH). ^13^C NMR (125 MHz, DMSO-*d*_6_, δ, ppm): 35.72 (CCH_2_N), 58.65 (OCHN), 115.5–157.0 (aromatic), 160.5 (C=O).

#### 2.1.4. PTyr-TPA Polypeptide

A solution of TPA-NH_2_ (0.16 g, 0.46 mmol) in DMF (6 mL) was added to a solution of Tyr-NCA (6.00 g, 22.7 mmol) in DMF (40 mL) that had been stirred for 30 min. After stirring the mixture for 72 h at 0 °C, the polypeptide was recovered through precipitation in Et_2_O; after dissolving it in CH_3_OH, it was recovered through precipitation in Et_2_O again. The product was then purified several times using CH_3_OH/Et_2_O cosolvents to obtain a pure gray powder that was dried at 50 °C under high vacuum. FTIR (cm^−^^1^): 3281 (NH), 1640, 1512, 720, 669 (Ar). ^1^H NMR (500 MHz, DMSO-*d*_6_, δ, ppm): 2.90 (d, 2H, CH_2_), 4.42 (t, 1H), 6.60 (d, 2H, Ar), 6.93 (d, 2H, Ar), 7.94 (s, 1H, NH) 9.13 (s, 1H).

#### 2.1.5. PTyr-TPA/P4VP Blends

PTyr-TPA/P4VP blends were prepared by dissolving desired amounts of PTyr-TPA and P4VP in DMF solution; the mixtures were stirred for 3 days. The solvent was then evaporated at 90 °C under high vacuum for 3 days to ensure the removal of residual DMF.

### 2.2. Characterization

^1^H and ^13^C NMR spectra were recorded using an INOVA 500 instrument (McKinley Scientific, Sparta, NJ, USA); DMSO-*d*_6_ was the solvent and tetramethylsilane (TMS) was the external standard. FTIR spectra were recorded using a Bruker Tensor 27 FTIR spectrophotometer (Bruker, Billerica, MA, USA); a KBr disk was used to prepare the sample, which was sufficiently thin to obey the Beer–Lambert law; the spectral resolution was 4 cm^−1^. Mass spectra were recorded using a Bruker Daltonics Autoflex MALDI-TOF mass spectrometer (Bruker, Billerica, MA, USA). The following voltage parameters were employed: ion source 1, 19.06 kV; ion source 2, 16.61 kV; lens, 8.78 kV; reflector 1, 21.08 kV; reflector 2, 9.73 kV. In addition, 2,5-dihydroxybenzoic acid (DHB) was used as a matrix for MADLI-TOF mass spectroscopy. UV–Vis absorption spectra were recorded using an optics DT 1000 CE 376 spectrophotometer (Prior, London, UK). PL analyses were performed using a LabGuide X350 fluorescence spectrometer (Prior, London, UK), with a 450-W Xe lamp as the continuous light source. The wavelength of excitation is 330 nm used in this study. DSC thermograms were measured under N_2_ atmosphere using a TA Q-20 differential scanning calorimeter (TA Instrument, New Castle, DE, USA); the sample (ca. 7–10 mg) was placed into a sealed aluminum sample pan and heated from room temperature to 250 °C at a heating rate of 20 °C/min. WAXD analyses were performed using the BL17A1 wiggler beamline at the National Synchrotron Radiation Research Center (NSRRC), Taiwan; the wavelength (*λ*) was 1.33 Å.

## 3. Results and Discussion

### 3.1. Synthesis of TPA-NH_2_

**Scheme 1:**
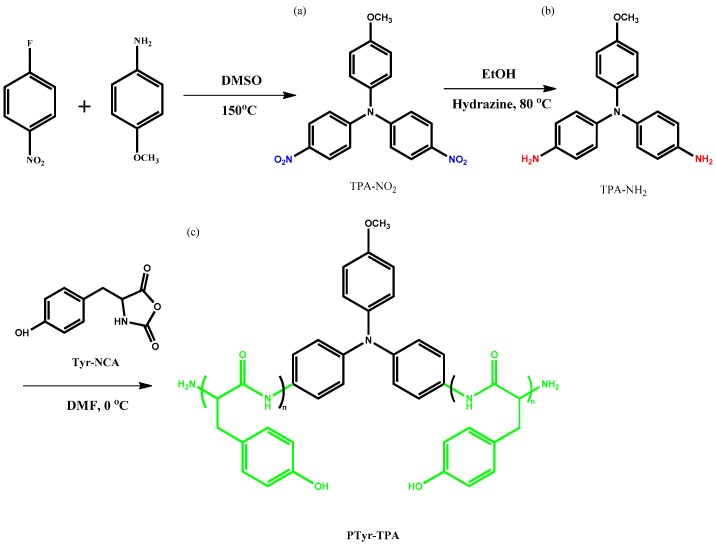
Synthesis of: (**a**) TPA-NO_2_; (**b**) TPA-NH_2_; and (**c**) PTyr-TPA.

Scheme 1 displays the two-step synthesis of the triphenylamine (TPA) derivative TPA-NH_2_ [44]. The condensation of *p*-anisidine with 1-fluoro-4-nitrobenzene in DMSO formed the dinitro-containing TPA compound, the nitro group of which was then reduced with N_2_H_4_ in absolute EtOH in the presence of 10 wt % Pd/C as the catalyst, giving the TPA-NH_2_ compound.

**Figure 1:**
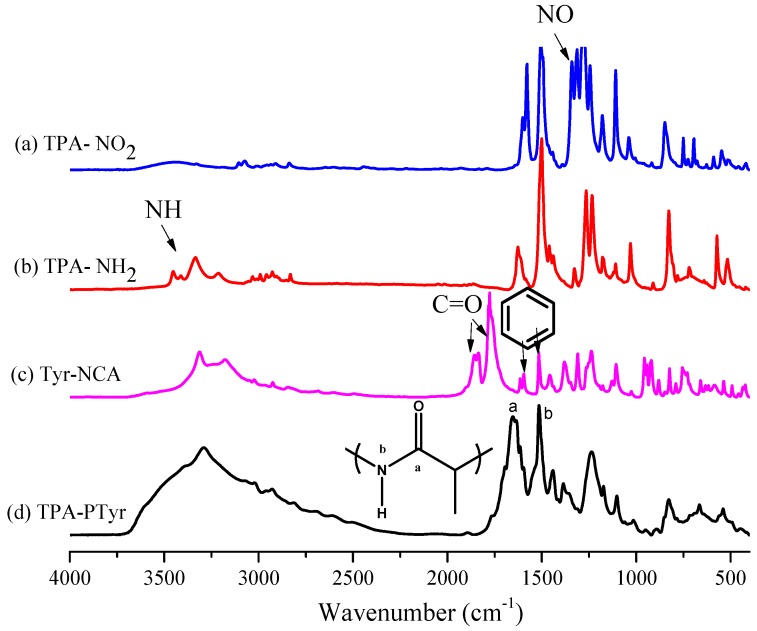
FTIR spectra, recorded at room temperature, of: (**a**) TPA-NO_2_; (**b**) TPA-NH_2_; (**c**) Tyr-NCA monomer; and (**d**) PTyr-TPA.

**Figure 2:**
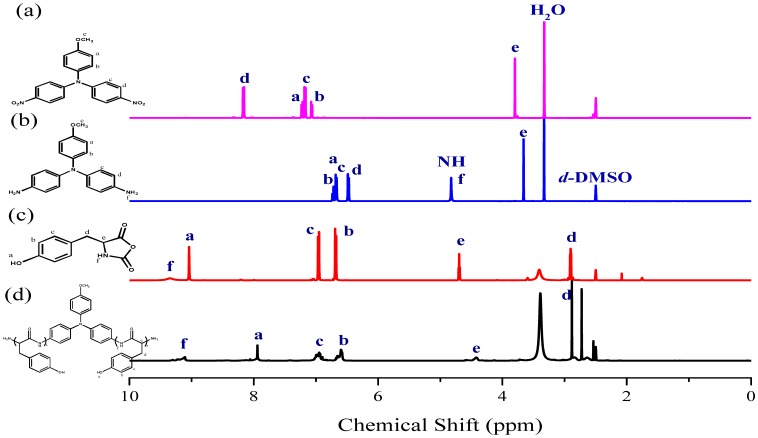
^1^H NMR spectra of: (**a**) TPA-NO_2_; (**b**) TPA-NH_2_; (**c**) Tyr-NCA monomer; and (**d**) PTyr-TPA.

**Figure 3:**
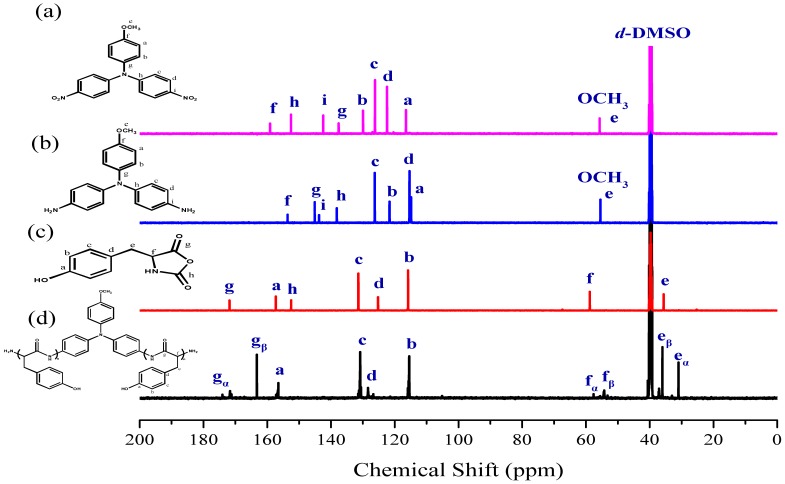
^13^C NMR spectra of: (**a**) TPA-NO_2_; (**b**) TPA-NH_2_; (**c**) Tyr-NCA monomer; and (**d**) PTyr*TPA.

Figure 1a,b displays the FTIR spectra of TPA-NO_2_ and TPA-NH_2_, respectively. The spectrum of TPA-NO_2_ features a signal at 1340 cm^−1^ corresponding to the NO_2_ group. After hydrogenation, absorption peaks were present for TPA-NH_2_ at 3456 and 3333 cm^−1^, corresponding to asymmetric and symmetric NH_2_ stretching, respectively. Figure 2a,b present the respective ^1^H NMR spectra of these two TPA derivatives. The signals for the aromatic protons appeared in the range from 6.47 and 8.10 ppm, dependent on whether a nitro or amino substituent was present. For TPA-NO_2_ (Figure 2a), a signal appeared at 3.80 ppm for the OCH_3_ unit; after hydrogenation (Figure 2b), two peaks were observed for TPA-NH_2_ at 3.66 ppm (slightly upfield OCH_3_ group) and 4.82 ppm (NH_2_ group). The ^13^C NMR spectra of TPA-NO_2_ and TPA-NH_2_ (Figure 3a,b, respectively) each feature signals for eight aromatic carbon nuclei in the range from 114 to 159 ppm and one for the OCH_3_ unit at 55.72 ppm; the chemical shifts of those aromatic carbon nuclei were strongly dependent on the substituent groups of these two TPA compounds. For example, peak **f** was shifted from 159.0 ppm for TPA-NO_2_ to 153.0 ppm for TPA-NH_2_. These spectra all suggest the successful synthesis of highly pure TPA-NH_2_.

### 3.2. Synthesis of PTyr-TPA through NCA-ROP Polymerization

**Figure 4:**
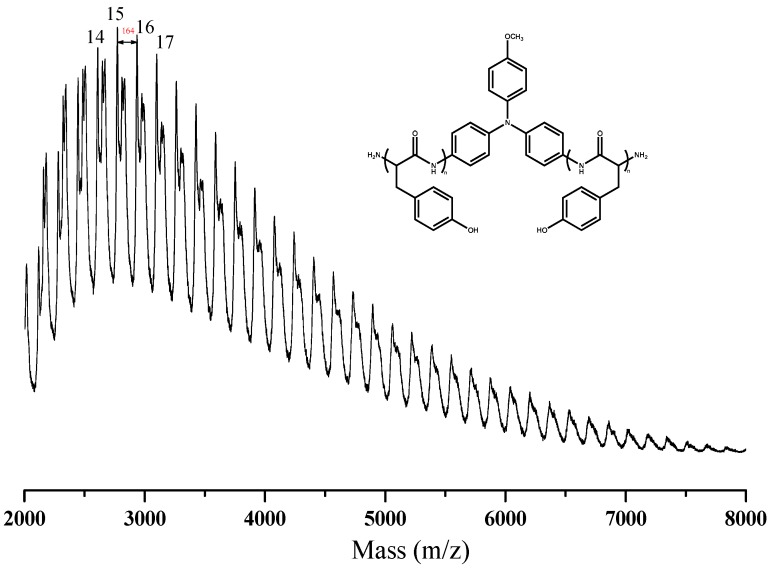
MALDI-TOF mass spectral analyses for PTyr-TPA.

The targeted polypeptide PTyr-TPA was synthesized readily through ring-opening polymerization of an α-amino acid NCA monomer at room temperature, using TPA-NH_2_ as the initiator. We synthesized the Tyr-NCA monomer using a previously reported procedure and confirmed its structure using FTIR and NMR spectroscopy. Figure 1c,d presents the FTIR spectra of the Tyr-NCA monomer and PTyr-TPA. The spectrum of Tyr-NCA features two absorptions at 1772 and 1850 cm^−1^, characteristic of anhydride C=O stretching. In addition, the absorption peak at 3290 cm^−^^1^ is due to the NH stretching and the absorption peaks at 1612 and 1512 cm^−^^1^ correspond to aromatic rings with phenolic OH units. After ring opening polymerization of Tyr-NCA, the anhydride absorptions disappeared and new signals appeared at 1653 and 1546 cm^−1^, corresponding to the amide I and amide II vibrations of the peptide bonds of PTyr-TPA. The detailed secondary structure of amide I absorption peak is discussed later. The ^1^H NMR spectrum of Tyr-NCA (Figure 2c) features a singlet at 9.04 ppm corresponding to the phenolic OH group, a singlet at 9.34 ppm assigned to the NH group, two doublets at 6.66 and 6.79 ppm for the protons of the CH units in the aromatic ring, a doublet at 2.49 ppm representing alkyl CH_2_ protons, and a triplet at 4.66 ppm arising from the alkyl CH proton. The ^1^H NMR spectrum of PTyr-TPA in Figure 2d reveals the typical signal for phenolic OH protons at 9.10 ppm in addition to a singlet at 7.93 ppm for the NH proton, a singlet at 4.42 ppm for the COCHNH backbone, a signal at 2.88 ppm corresponding to the CH_2_ protons, and two signals at 6.58 and 6.94 ppm corresponding to the aromatic protons. Figure 3c displays the ^13^C NMR spectrum of Tyr-NCA; the signals for the C=O nuclei appeared at 152.02 and 171.54 ppm, the phenolic OH carbon atom at 157.44 ppm, the α-carbon atom (NHCOC) at 58.88 ppm, and the benzyl carbon atom at 35.07 ppm. The ^13^C NMR spectrum of PTyr-TPA (Figure 3d) features signals for the amide carbon (CONH) atoms at 163.16 and 171.78 ppm in their β-sheet and α-helical conformations, respectively; furthermore, the α-carbon atoms (NHCOC) of the backbone were represented by signals at 54.44 and 57.53 ppm, again due to the β-sheet and α-helical conformations, respectively. Figure 4 presents the MALDI-TOF mass spectrum of PTyr-TPA; the mass difference (*m*/*z*) between each pair of adjacent peaks was 164 Da, equal to the repeat unit of PTyr. In addition, each peak is the molecular weight from 302 Da (the molecular weight of TPA-NH_2_) + 164*n* (*n* = degree of polymerization of PTyr). Taken together, the FTIR, NMR, GPC and MALDI-TOF mass spectra confirmed our successful synthesis of PTyr-TPA (GPC: *M*_n_ = 3500 Da, PDI = 1.10, MALDI-TOF MS: *M*_n_ = 3506 Da; PDI = 1.10).

### 3.3. AIE Behavior

**Figure 5:**
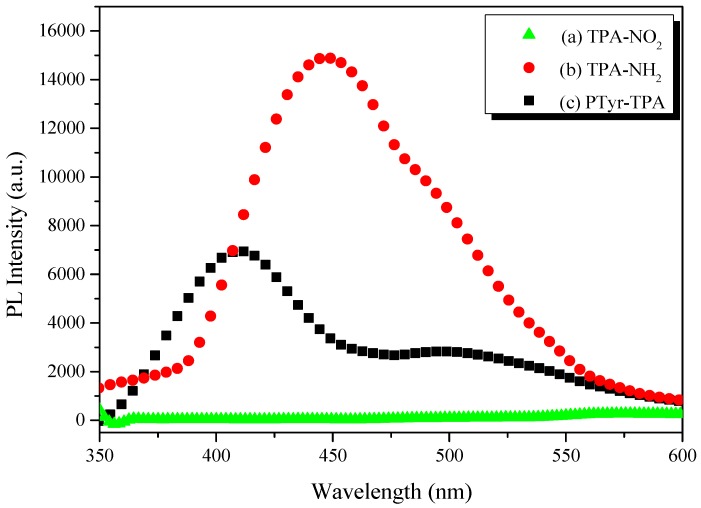
PL spectra (excitation wavelength: 330 nm) of: (**a**) TPA-NO_2_; (**b**) TPA-NH_2_; and (**c**) PTyr-TPA in the solid state.

Figure 5 displays the fluorescence emission spectra of TPA-NO_2_, TPA-NH_2_, and PTyr-TPA in the solid state. Although TPA-NO_2_ did not provide any clear emission peaks, TPA-NH_2_ exhibited two fluorescence emission signals at 447 (monomer emission) and 491 (excimer emission) nm. Figure 5 reveals that the PTyr-TPA polypeptide exhibited its emission at 410 nm (i.e., hypsochromic shift). Most organic emissive compounds are highly emissive in solution, but display quenched emission in concentrated solutions and in the solid (aggregate) state because of non-covalent interaction (e.g., π-stacking), so-called ACQ. Nevertheless, Tang et al. have prepared many compounds that exhibit strong emission in the aggregated state, related to the AIE effect [39,40,41]. We carefully studied the possible AIE behavior of TPA-NH_2_ and PTyr-TPA by using concentration and solvent pair effects.

**Figure 6:**
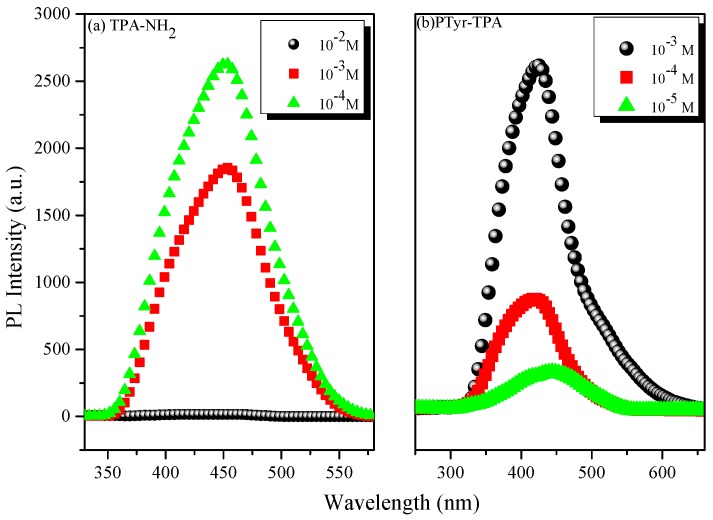
PL spectra (excitation wavelength: 330 nm) of: (**a**) TPA-NH_2_; and (**b**) PTyr-TPA as solutions in THF.

**Figure 7:**
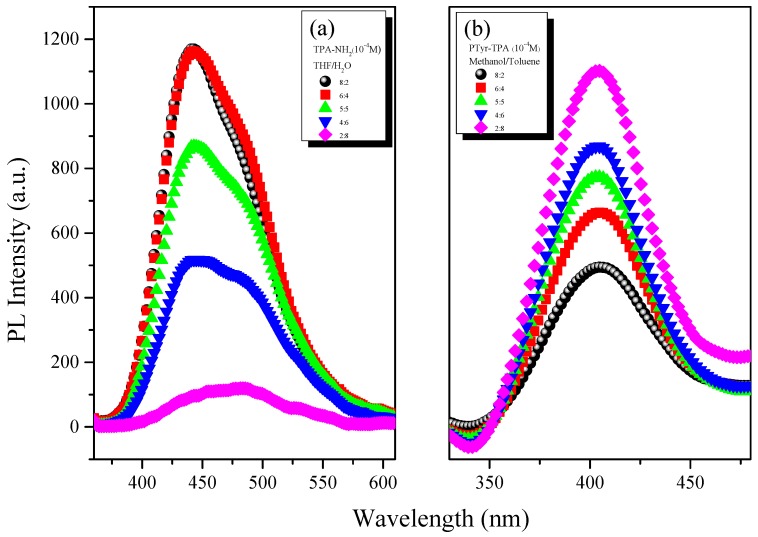
PL spectra (excitation wavelength: 330 nm) of: (**a**) TPA-NH_2_ in THF/H_2_O; and (**b**) PTyr-TPA in CH_3_OH/toluene at a concentration of 10^−4^ M.

**Figure 8:**
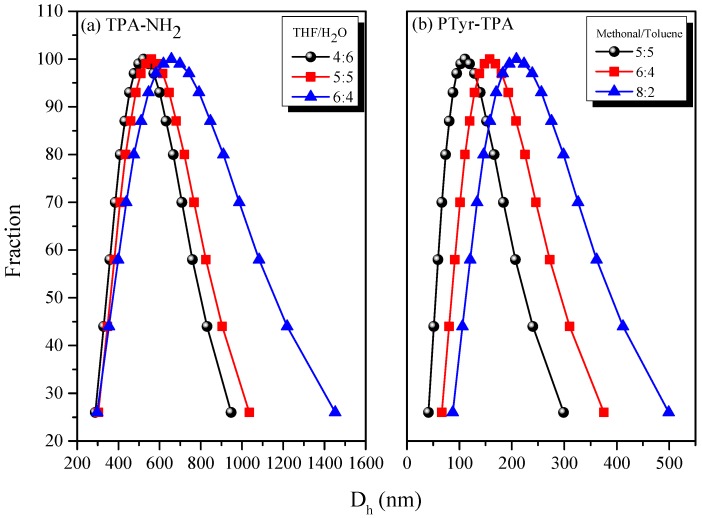
DLS analyses of: (**a**) TPA-NH_2_ in THF/H_2_O; and (**b**) PTyr-TPA in CH_3_OH/toluene, with various amounts of the poor solvents.

Figure 6 displays the fluorescence emissions of TPA-NH_2_ and PTyr-TPA in THF at various concentrations. Figure 6a reveals that the intensity of the emission decreased upon increasing the concentration of TPA-NH_2_ in THF. This concentration-quenched emission occurred for TPA-NH_2_ because of intramolecular π-stacking through excimer formation. In contrast, PTyr-TPA displayed (Figure 6b) higher emission in THF when its concentration was 10^−3^ M than it did in dilute solution (10^−5^ M). Accordingly, we conclude that the emission of TPA-NH_2_ transformed from ACQ to AIE behavior after incorporation into the rigid main chain of PTyr. To further examine the ACQ behavior of TPA-NH_2_, we dissolved it in THF/H_2_O (solvent/poor solvent); Figure 7a reveals that TPA-NH_2_ emitted strongly at 441 nm when in a dilute solution (ca. 10^−4^ M) in THF. The intensity of the fluorescence emission decreased, however, upon increasing the concentration of H_2_O. We did not use THF as the good solvent for PTyr-TPA to avoid any possible hydrogen bonding interactions between the phenolic OH groups of PTyr and the ether group of the solvent. Interestingly, dissolving TPyr-TPA in MeOH (good solvent) and changing the concentration of toluene (poor solvent) caused the intensity of the fluorescence emission to increase upon increasing the concentration of toluene up to 80%, because of aggregation (Figure 7b), consistent with AIE behavior. We also examined these two systems using dynamic light scattering (DLS, Figure 8). Both systems exhibited smaller particle sizes upon increasing the concentration of the poor-solvent (H_2_O for TPA-NH_2_; toluene for PTyr-TPA), because of aggregation and shrink the PTyr chain as expected. Based on these results, we conclude that TPA-NH_2_ is an ACQ compound, whereas the new PTyr-TPA fluorescent polypeptide displayed AIE behavior.

### 3.4. Physical Properties of PTyr-TPA/P4VP Blends

#### 3.4.1. Thermal Properties of Binary PTyr-TPA/P4VP Blends

**Figure 9:**
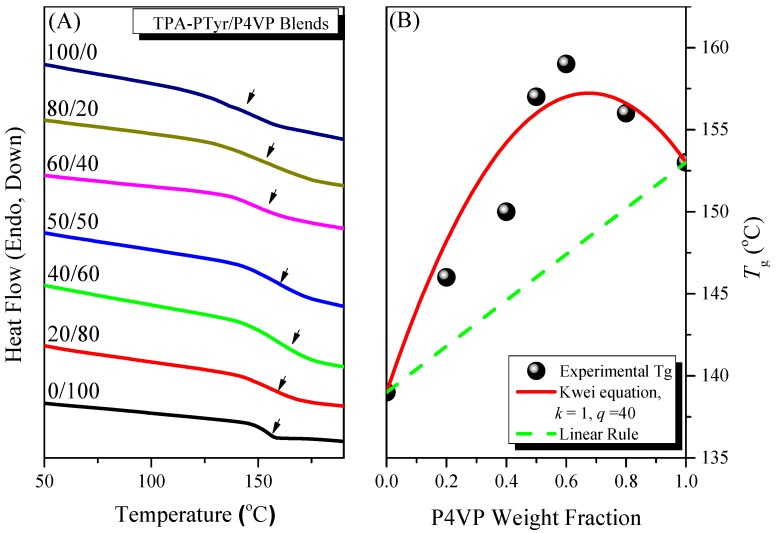
(**A**) DSC thermograms of miscible PTyr-TPA/P4VP blends (second heating scan); and (**B**) the *T_g_* behavior of the miscible PTyr-TPA/P4VP blends and that predicted by the Kwei equation.

DSC was a convenient method for thermal analysis of binary PTyr-TPA/P4VP blends. In general, a miscible blend system displays a single glass transition temperature (*T_g_*). Figure 9A displays DSC thermograms of our miscible PTyr-TPA/P4VP blend system, stabilized through hydrogen bonding interactions. In a previous study, we found that separated random coils of PTyr formed when blended with P4VP in DMF solution [26]. Figure 9A reveals that the value of *T_g_* of pure PTyr-TPA (139 °C) was lower than that of the pure linear PTyr (155 °C), due to the non-coplanar and bowl-shaped TPA unit inducing an increase in the free volume of the former. Single-*T_g_* behavior was evident in the PTyr-TPA/P4VP blends, suggesting complete miscibility. In addition, the values of *T_g_* in the PTyr-TPA/P4VP blends increased upon increasing the concentration of P4VP because of intermolecular hydrogen bonding between the phenolic OH groups of PTyr-TPA and the pyridine groups of P4VP. In addition, the broad *T_g_* is usually observed for the miscible blend due to larger self-concentration and length scale [45]. Moreover, we calculate values of *k* and *q* of 1 and 40, respectively, for the PTyr-TPA/P4VP blend, based on the Kwei Equation [46]:

(1) $$T_{g}=\frac{W_{1}T_{g1}+kW_{2}T_{g2}}{W_{1}+kW_{2}}+qW_{1}W_{2}$$

where *T_g_* is the corresponding glass transition temperature of each component about P4VP and Pty, *W*_i_ represents the weight fraction, and *k* and *q* are the fitting constant. This positive value of *q* suggests that the intermolecular interactions in the PTyr-TPA/P4VP blend were stronger than those for the self-association of the phenolic OH units of PTyr-TPA [47,48,49,50].

#### 3.4.2. Secondary Structure and Hydrogen Bonding of PTyr-TPA/P4VP Blends

**Figure 10:**
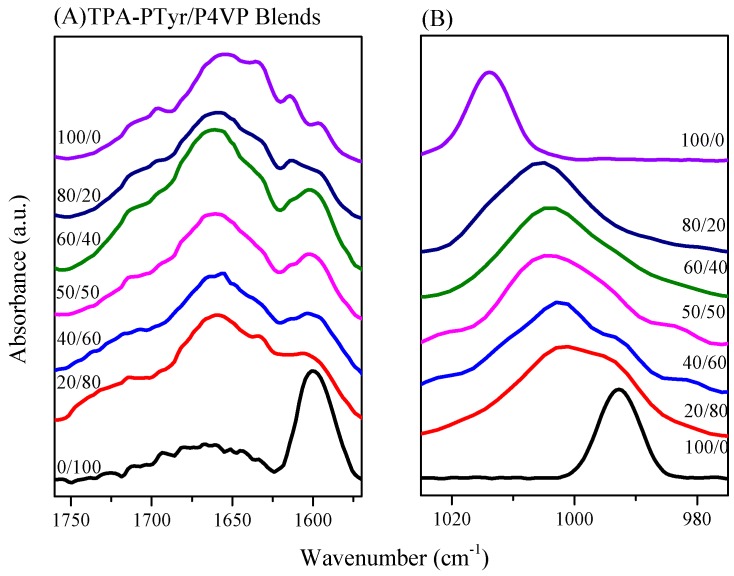
FTIR spectra, recorded at room temperature, of the miscible PTyr-TPA/P4VP blends in the regions: (**A**) 1760–1570 cm^−1^ (amide I absorption); and (**B**) 1030–970 cm^−1^ (pyridine unit absorption).

**Figure 11:**
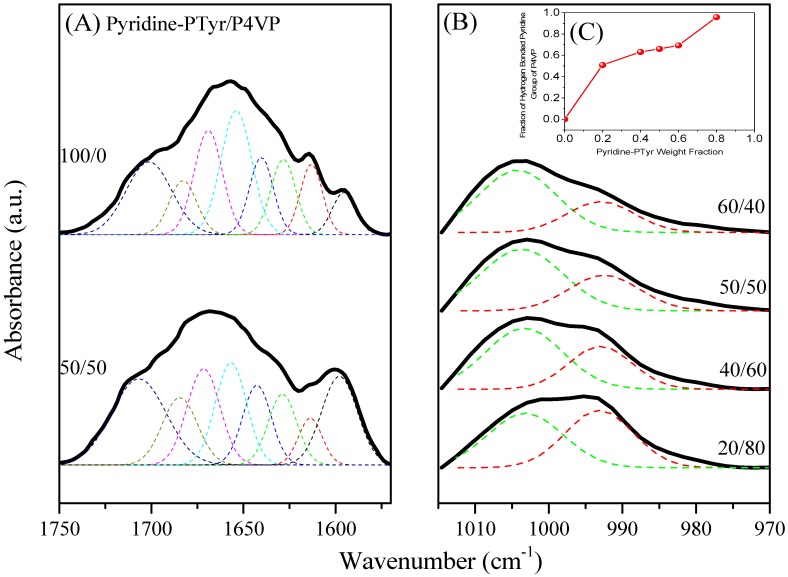
Curve fitting of the data in Figure 10 for the miscible PTyr-TPA/P4VP blends for the: (**A**) amide I absorption; (**B**) pyridine unit absorption; and (**C**) fraction of hydrogen-bonded pyridine units upon increasing the content of PTyr-TPA.

FTIR spectral analysis was a rapid and facile method for examining the hydrogen bonding interactions and secondary structures of the peptide conformations in the PTyr-TPA/P4VP blends. Figure 10 shows that the amide I absorption was located near 1650 cm^−1^ along with absorption signals near 1630 cm^−1^ and for the α-helical and β-sheet conformations, respectively [50]. Papadopoulous et al. reported that the α-helical and β-sheet conformations were strongly correlated to the degree of polymerization (DP) of PBLG, with the α-helical conformation favored when the DP was greater than 18 while both conformations were present for DPs of less than 18 [51]. As displayed in Figure 10A, we observed eight major absorptions for the pure PTyr-TPA: for the aromatic ring vibrations from PTyr (1595 and 1612 cm^−1^), the α-helical conformation (1655 cm^−1^), the β-turn conformation (1671 cm^−1^), the β-sheet conformation (1630 cm^−1^), and the random coil conformation (1641, 1687, and 1705 cm^−1^) [52]. Figure 11A summarizes the results of curve fitting using the second-derivative technique. The fractions of α-helical and β-sheet conformations both decreased and the fraction of the random coil conformations increased when blending with 50 wt % P4VP. For example, the fraction of β-sheet conformations decreased from 19.4% for the pure PTyr-TPA to 9.48% for the blend with 50 wt % P4VP. In addition, we investigated the hydrogen bonding interactions of the PTyr-TPA/P4VP blends (Figure 10B). The signal for the free pyridine units of P4VP was located at 993 cm^−1^; it shifted to 1005 cm^−1^ when blended with PTyr-TPA, representing the pyridine groups hydrogen-bonded with the phenolic OH groups of PTyr-TPA [46,47,48]. We could separate these two peaks to calculate the fraction of hydrogen-bonded pyridine units, by digitally subtracting the signal for the pure PTyr-TPA at 1013 cm^−1^, according to the weight fraction of PTyr-TPA in the blend system (Figure 11B). The fraction of hydrogen-bonded pyridine units increased upon increasing the concentration of PTyr-TPA, as displayed in Figure 11C (the inset of Figure 11B).

**Figure 12:**
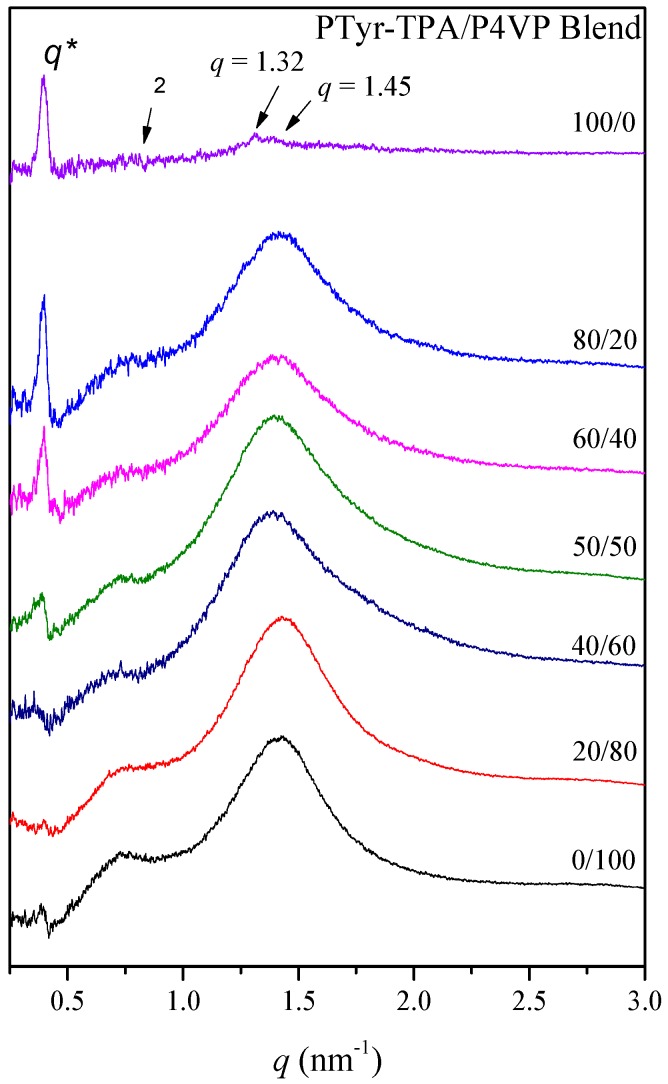
WAXD analyses of the miscible PTyr-TPA/P4VP blends.

Figure 12 presents the WAXD data used to determine the change in the secondary structure for the PTyr-TPA/P4VP blends. These data suggested a β-sheet conformation for the pure PTyr-TPA (DP = 11). The first diffraction peak appeared at a value of *q* of 0.4 nm^−1^ (*d* = 1.57 nm), correlated to the distance within the antiparallel β-pleated sheet of the backbones. In addition, the diffraction peak at a value of *q* of 1.45 nm^−1^ represents the repeated residues of the PTyr backbone (*d* = 0.445 nm), with the signal at a value of *q* of 1.32 nm^−1^ (*d* = 0.475 nm) originating from the intermolecular distance between the adjacent PTyr backbones within one lamella [53]. Increasing the P4VP compositions in the PTyr-TPA/blends caused the β-sheet conformation to disappear, with the patterns becoming a broad amorphous halo representing the random coil conformation, consistent with our previous findings [26,27].

#### 3.4.3. Emission Properties of PTyr-TPA/P4VP Blends

**Figure 13:**
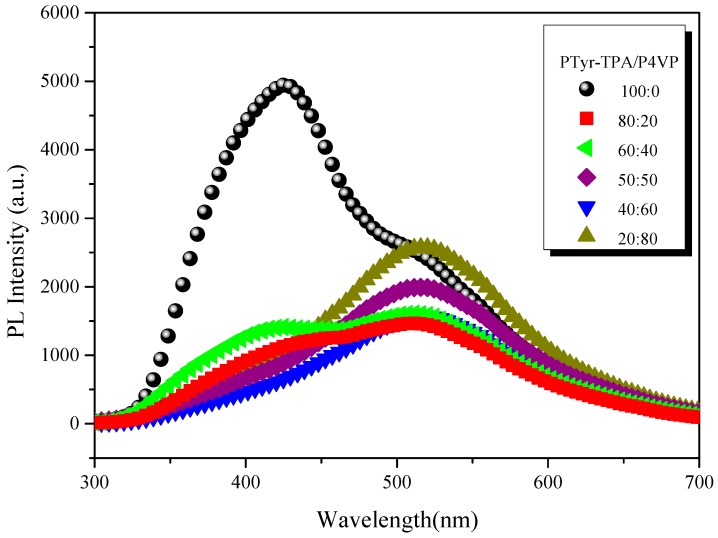
PL spectra (excitation wavelength: 330 nm) of PTyr-TPA/P4VP blends in the solid state.

**Figure 14:**
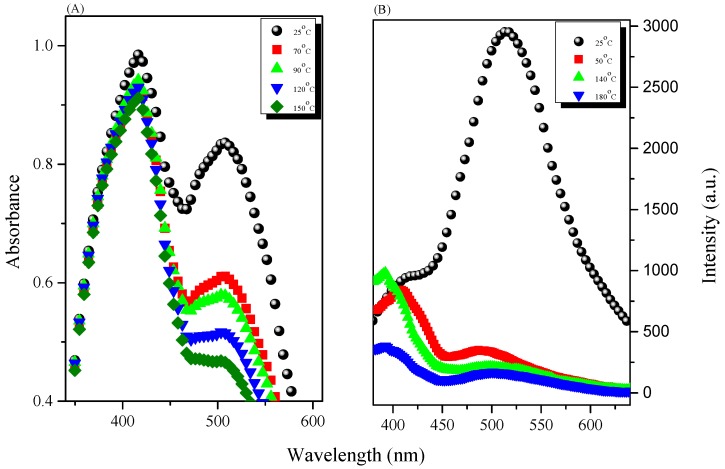
UV (**A**); and PL (**B**) spectra of the PTyr-TPA/P4VP = 20/80 blend in the solid state, recorded at various temperatures.

**Figure 15:**
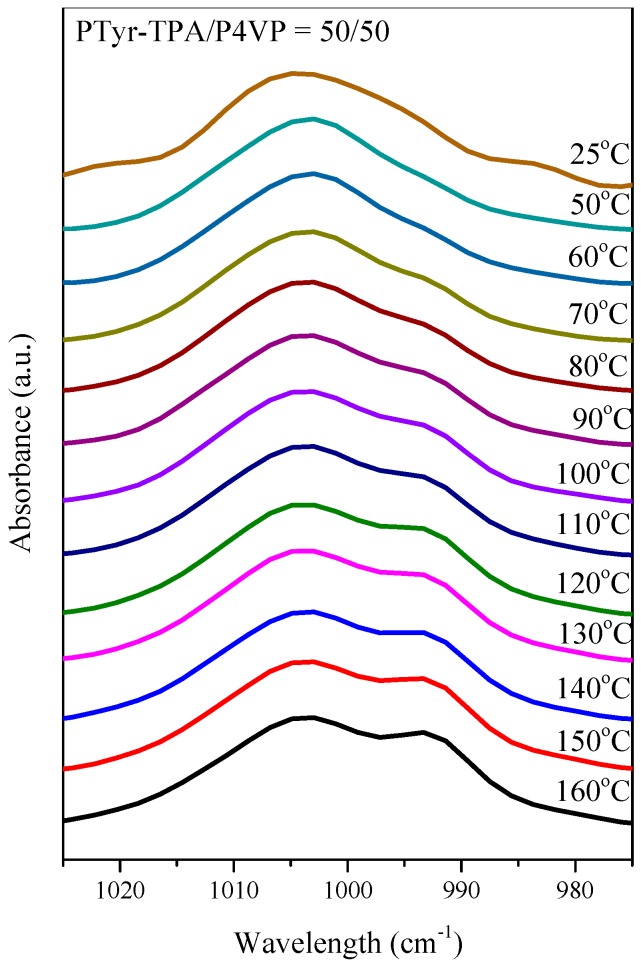
FTIR spectra of the PTyr-TPA/P4VP = 20/80 blend in the solid state, recorded at various temperatures.

Tang et al. proposed that the AIE mechanism was due to a mechanism involving restricted intramolecular rotation (RIR) [39,40,41]. We found that PTyr-TPA exhibited AIE behavior and a high fluorescence emission intensity. Blending with P4VP caused the PTyr-TPA emission intensity to decrease, with the maximum peak bathochromic shift occurring from 426 to 522 nm (Figure 13). We attribute this behavior to the hydrogen bonding between the phenolic OH groups of PTyr-TPA and the pyridine groups of P4VP and the release of the RIR of the TPA unit in the polypeptide center. When the content of P4VP reached 80 wt %, the corresponding fluorescence intensity increased, due to the RIR mechanism for TPA unit. The solid state quantum efficiencies (*Φ*_f_) of the PTyr-TPA/P4VP blend systems containing 0, 20, 40, 50, 60, and 80 wt % of P4VP were 0.4, 0.321, 0.366, 0.36, 0.352, and 0.287, respectively. To confirm that the peak at 522 nm was due to hydrogen bonding between the phenolic OH groups of PTyr-TPA and the pyridine groups of P4VP, we investigated the UV and PL spectra of the PTyr-TPA/P4VP = 20/80 blend at various temperatures (Figure 14). The intensity of the signal at 522 nm, due to the excimer emission, decreased upon increasing the temperature, as displayed in Figure 14B, indicating that the fraction of hydrogen-bonded pyridine units decreased accordingly, consistent with the FTIR spectral analyses in Figure 15, where the fraction of hydrogen-bonded pyridine units (evidenced by the signal at 1005 cm^−1^) decreased upon increasing the temperature.

## 4. Conclusions

We have used TPA-NH_2_ as an initiator to synthesize a PTyr-TPA homopolymer through NCA living ring opening polymerization. The chemical structures of these compounds were confirmed using NMR, FTIR, and MALDI-TOF mass spectra. PL analyses indicated that TPA-NH_2_ behaved as an ACQ compound, whereas it became a strong AIE compound after attachment to the rigid-rod conformation of PTyr because of RIR mechanism. DSC analyses revealed single-*T_g_* behavior for the miscible PTyr-TPA/P4VP blends. The PL emission intensity of PTyr-TPA decreased upon blending with P4VP, due to the release of the RIR mechanism for the TPA unit in the PTyr, resulting from hydrogen bonding between the pyridine groups of P4VP and the phenolic OH groups of PTyr-TPA. Furthermore, WAXD analyses revealed that the chains of PTyr-TPA transformed from a β-sheet conformation to random coils after blending with P4VP.
